# Perspectives on the ketogenic diet as a non-pharmacological intervention for major depressive disorder

**DOI:** 10.47626/2237-6089-2024-0932

**Published:** 2025-12-05

**Authors:** Jade Shelp, Cristiano Chaves, Alexander Terpstra, Elena Koning, Fabiano A. Gomes, Jennifer Fabe, Vitor Breda, Nicole Laurent, Christopher Palmer, Elisa Brietzke

**Affiliations:** 1 Queen's University School of Medicine Department of Psychiatry Kingston ON Canada Department of Psychiatry, Queen's University School of Medicine, Kingston, ON, Canada.; 2 Queen's University Centre for Neuroscience Studies (CNS) Kingston ON Canada Centre for Neuroscience Studies (CNS), Queen's University, Kingston, ON, Canada.; 3 St. Joseph's Healthcare Hamilton Hamilton ON Canada St. Joseph's Healthcare Hamilton, Hamilton, ON, Canada.; 4 McMaster University Department of Psychiatry and Behavioural Neurosciences Hamilton ON Canada Department of Psychiatry and Behavioural Neurosciences, McMaster University, Hamilton, ON, Canada.; 5 McMaster Children's Hospital Hamilton ON Canada McMaster Children's Hospital, Hamilton, ON, Canada.; 6 Western University London Health Sciences Centre Department of Psychiatry London ON Canada Department of Psychiatry, London Health Sciences Centre, Western University, London, ON, Canada.; 7 Inc DBA Mental Health Keto Family Renewal Vancouver WA United States Family Renewal, Inc DBA Mental Health Keto, Vancouver, WA, United States.; 8 McLean Hospital Belmont MA USA McLean Hospital, Belmont, MA, USA.; 9 Harvard Medical School Boston MA USA Harvard Medical School, Boston, MA, USA.

**Keywords:** Major depressive disorder, ketogenic diet, ketosis, metabolism, nutrition

## Abstract

**Objective:**

Major depressive disorder (MDD) is a prevalent mood disorder characterized by persistent low mood and anhedonia, significantly impacting cognitive function and daily living. Despite available pharmacological treatments, nearly one-third of individuals with MDD do not achieve adequate symptom relief with conventional treatments. The ketogenic diet (KD), a high-fat, low-carbohydrate diet that induces ketosis, has emerged as a potential non-pharmacological intervention for MDD. The objective of this study was to provide a comprehensive perspective on the current knowledge and gaps regarding the potential antidepressant effect of the KD, emphasizing its safety, efficacy, and mechanistic pathways.

**Methods:**

This narrative review synthesizes data from preclinical and clinical studies on the effects of KD on mood, cognitive function, and its potential as an antidepressant. Mechanistic insights from animal and human studies are explored to elucidate possible pathways through which KD may exert its effects on MDD.

**Results:**

Evidence from animal models suggests that KD may reduce depressive-like behaviors and improve cognitive function. Preliminary human studies, including case reports and observational studies, indicate potential benefits such as mood stabilization, increased energy, and reduced depression severity. Proposed mechanisms include immune-inflammatory regulation, correction of mitochondrial dysfunction, and neurotransmitter modulation. However, key gaps remain, particularly regarding the therapeutic window, long-term efficacy, and specific mechanisms of action in MDD.

**Conclusions:**

KD represents a promising avenue for further investigation as a non-pharmacological treatment of MDD. Further research is needed to establish its clinical utility, identify predictors of response, and assess its feasibility as a treatment option for MDD.

## Introduction

Major depressive disorder (MDD) or unipolar depression is a prevalent and potentially chronic condition with both brain and systemic morbidity.^1^ Its causes include genetic and environmental factors operating through numerous mechanisms including neurotransmission, neuroplasticity, energetic metabolism, and immuno-inflammatory pathways. Numerous conventional treatments and interventions are available, mainly targeting the monoaminergic neurotransmitter system.^2^ However, they are ineffective in over one-third of MDD patients.^3^ Consequently, there is a demand for novel treatments that are effective, safe and affordable, such as metabolic non-pharmacological treatments.^4^

The ketogenic diet (KD) has gained attention as a potential option for the treatment of mental disorders.^5^ The KD is characterized by high fat intake and low carbohydrate consumption, leading to a metabolic state of ketosis. In this state, the body consumes fat as its primary energy source due to the absence of carbohydrates and the inability to complete glycolysis, producing higher levels of circulating ketones.^6^ Promising studies of animal models for depression have shown that the KD may exert antidepressant-like effects.^7,8^ Observational studies have also demonstrated an antidepressant and mood stabilizing effect from the KD in case reports and case series.^9,10^

Considering these promising preliminary results and the limitations of current antidepressant medications, further research is needed to allow the implementation of KD in clinical settings for MDD treatment. In this piece, we provide a perspective of current knowledge and gaps in understanding regarding the potential antidepressant effect of the KD. This work provides a guide for researchers, clinicians, and stakeholders in the field of psychiatry and contributes to the refinement of KD as an evidence-based intervention for MDD. This paper builds on the information presented by similar articles^11,12^ by exploring further into the existing knowledge and identifying the gaps in the current literature.

## What do we know about the ketogenic diet that supports its antidepressant effect?

### The ketogenic diet is overall safe

Although the KD has long been used for weight loss and the treatment of epilepsy, potential health outcomes should be carefully considered before implementation.^13^ Research in populations with epilepsy has shown that short-term side effects of the KD may include fatigue, headache, nausea, and constipation, while long-term effects can encompass hepatic steatosis, kidney stones, hypoproteinemia, and vitamin deficiency.^13^ Another safety concern regarding the KD is hypercholesterolemia, in which an individual may develop high cholesterol following the diet. Although an individual's low-density lipoprotein (LDL) cholesterol response to dietary changes varies, the monitoring of blood lipid profile is important for all individuals on the KD,^14^ Elevations in LDL cholesterol are almost exclusively experienced by lean individuals,^15^ and their implications on cardiovascular health remain unknown.^16^ Most side effects of the KD occur during the first few weeks of the diet's initiation and resolve themselves.^6,13^ If these side effects persist, carbohydrates are periodically reintroduced into the diet or lipid-lowering medications are used.^17^ It is important to note that the KD may not be suitable for all patients. Although the KD has been shown to improve insulin sensitivity, patients with diabetes on insulin or using oral hypoglycemic agents may experience severe hypoglycemia during the initiation of the diet. Further, the KD is not suggested for individuals with liver failure, pancreatitis and metabolic disorders related to fat consumption such as primary carnitine deficiency.^13^ It is also possible that weight loss induced by the KD may be an adverse effect for individuals with MDD, in which changes to diet and nutritional status may impact the diagnosis and progression of the disorder, especially when employing unhealthy weight loss behaviors (e.g., skipping meals).^18,19^ These factors must be considered prior to clinical implementation and accounted for on a patient-to-patient basis.^20^ The term "ketogenic therapy" may be more appropriate to describe this intervention for MDD, as its primary goal is to achieve significant levels of ketosis, independent of any weight changes related to the diet. Notably, individuals may undergo a low-carbohydrate diet and experience weight loss without attaining significant levels of ketosis. Furthermore, some clinical trials for depression prescribe a ketogenic therapy using an isocaloric diet, mirroring approaches traditionally used to treat epilepsy.^21^

### The ketogenic diet is an effective treatment for epilepsy

Epilepsy is a disabling neurological condition often treated with anticonvulsant medications. Pharmacoresistant epilepsy occurs when a patient does not experience sustained seizure relief from adequate trials of at least two antiepileptic drugs, which occurs in nearly one-third of individuals with this condition.^22^ The KD has long been established as a treatment option for this clinical situation in children, adolescents, and adults in randomized controlled trials (RCTs) and meta-analyses. A 2020 Cochrane Review of the KD for pharmacoresistant epilepsy included 13 studies with 932 participants — 711 children and 221 adults.^23^ Compared to usual care, children assigned to the KD were three times more likely to experience seizure freedom and six times more likely to experience a reduction in seizure frequency. In adults assigned to the KD versus usual care, although none experienced seizure freedom, they were five times more likely to experience a reduction in seizures compared to usual care. However, the certainty of the evidence was judged to be low or very low due the limited number of studies and small sample sizes, highlighting the need for more research investigating therapeutic effects of the KD. To date, the strongest evidence to support the use of the KD for neurological conditions is in its treatment of pharmacoresistent epilepsy. A connection has been suggested between the pathophysiological mechanisms of epilepsy and MDD. The hippocampus plays an important role in the pathology of both seizure activity and mood disorders and may provide a link between the two. There is also a high prevalence of depression among individuals with epilepsy.^24^ Animal models of epilepsy have observed that decreased activity in serotonin, dopamine, norepinephrine, and GABA worsen seizure frequency and severity, and can be reversed by antidepressant medications. In addition, the pathogenic mechanisms of MDD include reduced activity of these neurotransmitters, further suggesting that MDD shares pathogenic mechanisms with epilepsy.^25^ In the epileptic brain, ketosis induces metabolic plasticity within neuronal cells, consequently changing the electrical activities of neurons. These changes to metabolites, such as ketone bodies, glucose and lactate, seems to elicit neuronal inhibition across many electrical regulators (e.g. ion channels, synaptic receptors, neurotransmitter transporters), creating the antiseizure effects of the KD.^26^ This mechanism may be of interest for the treatment of MDD as similar metabolic energy changes occur in the disorder.

### The ketogenic diet has a pro-cognitive effect

Among the most disabling symptoms of MDD are cognitive impairments, such as concentration and decision-making difficulties. These changes can interfere with daily functioning and may even persist after other symptoms of MDD have resolved.^1,27,28^ The impacts of KD on cognition and behaviour are important topics to explore to better understand the therapeutic effects of the diet. Studies with healthy aging animal models have demonstrated improved cognitive outcomes in response to KD, specifically in visual working memory and attention.^29,30^ Human trials have also been conducted to investigate the cognitive benefits of the KD. A previous RCT investigated the cognitive effects of KD for adolescents with pharmacoresistant epilepsy found that the KD group displayed a significant increase in word comprehension by the Peabody picture test scores whereas a care-as-usual group showed no significant changes.^31^ Another study comparing the cognitive effects of the KD to a gluten- and casein-free diet (GFCF) in children with autism spectrum disorder found that KD was associated with improvements in cognition and sociability components of the Childhood Autism Rating Scale and Autism Treatment Evaluation Test scales, compared to the GFCF group.^32^ The assessment of the KD as a treatment for neurodegenerative diseases, such as Alzheimer's disease (AD), is another area of interest. For instance, a randomized crossover-controlled trial was conducted comparing the effects of a modified KD on daily functioning and quality of life in AD patients relative to a control diet. The authors found that individuals in the KD group increased their competence in basic activities and an improved quality of life compared to the control group.^33^ In addition, the effect of oral administration of exogenous ketones to elicit hyperketonemia (elevated blood levels of ketones) was observed on a patient with AD. The study reported that the patient experienced an improvement in mood, affect, self-care and cognitive activity performance, as noted through conversation and interactions at higher levels than prior to the treatment.^34^ Collectively, these findings suggest that the KD may have beneficial effects on cognitive and behavioural functioning.

### There is preliminary evidence supporting an antidepressant effect of the ketogenic diet

There is limited, but promising, preliminary evidence of the efficacy of KD for depression from animal and human studies. A study using lipopolysaccharide and repeated social defeat stress animal models to measure the potential therapeutic effect of the KD on MDD and showed that the KD was associated with decreasing depressive-like behaviours.^23^ Other rodent studies demonstrated a reduction of anxiety and depression-like behaviours when the diet was administered.^7,8,35,36^

Case studies suggest potential benefits such as reduced severity of anxiety symptoms, mood stabilization, increased energy, and reduced severity of depression symptoms. A study investigating the effects of the KD on 31 inpatients with refractory mental illnesses, including 7 diagnosed with MDD, reported clinically significant improvements in depression symptoms.^9^ A case report of a 65-year-old woman with type-2 diabetes and MDD who underwent a 12-week intervention of the KD demonstrated an improvement in her depression symptoms.^10^ Another case report of a 70-year-old woman with MDD and schizophrenia indicated that she experienced increased energy and cessation of auditory and visual hallucinations after following the KD for 12 months.^37^ Interventional controlled clinical trials have not yet been conducted. These studies are limited by small sample sizes and heterogeneity in trial duration and methodology. Future research includes several new trials that will bring additional information on safety and efficacy. A randomized controlled open-label trial is being proposed to observe the clinical effects of a 3-month intervention of the Modified Atkins diet (MAD) on MDD patients ages 18-65 receiving standard treatment.^38^ Furthermore, additional clinical studies investigating this relationship are currently underway.^21,39-41^ This growing body of low-level evidence is a call for rigorous and larger clinical trials investigating the antidepressant potential of the KD.

## What are the main gaps in the knowledge about a potential antidepressant effect of the ketogenic diet?

### Level of ketosis necessary to elicit an antidepressant response is unknown

A critical outstanding question regarding the use of the KD for MDD is the level of ketones in the body that is necessary to elicit an antidepressant response. Beta-hydroxybutyrate (BHB) is the primary blood ketone that is used to measure the level of ketosis in the body through blood samples. In a maintained KD, a BHB concentration should remain within 0.5-8mmol/L, marking a state of physiological ketosis. For the treatment of children, clinicians recommend that the BHB levels remain within 4-6mmol/L, often achieved through a classic KD (3:1 or 4:1 ratio of fats to carbohydrates/protein).^42^ The standard KD typically limits carbohydrate intake to approximately 20-50 grams per day, with a diet consisting of foods rich in fat (e.g. olive oil, butter, nuts) and a smaller amount of protein-rich foods (e.g. meat, fish) as well as vegetables and fruits.^43,44^ However, it is unknown whether similar BHB levels are required to produce an antidepressant response or if lower BHB levels could alleviate depressive symptoms, allowing for a less restrictive KD approach.

For some individuals, the classic KD can be difficult to maintain for a sustained period. Less restrictive, alternative low carbohydrate/high fat diets have emerged, including the MAD (1:1 or 2:1 fats to carbohydrates and proteins) and the low-glycemic index diet (0.6:1 fats to carbohydrates).^45^ Studies have shown that they have similar efficacy as anticonvulsants.^46,47^ A recent study found a significant positive correlation over an 8-week period between daily ketone levels and ecological momentary assessments of mood (r = 0.21, p < 0.001) and energy (r = 0.19, p < 0.001).^48^ Additionally, there was a negative correlation between ketone levels and both impulsivity (r = −0.22, p < 0.001) and anxiety (r = −0.18, p < 0.001). Therefore, it is important for future studies to investigate the therapeutic range of ketone levels necessary to elicit antidepressant effects.

### The long-term efficacy, tolerability, and feasibility of KD in MDD are unknown

One of the main limitations of the literature in the use of KD for non-epileptic conditions is the short-term follow-up periods. Most studies followed participants for four to 12 weeks, except for two case reports that observed individuals with comorbid MDD and schizophrenia.^49,50^ The follow-up period for previous studies is not representative of the illness course for most individuals with MDD. In addition, the potential of KD as a maintenance treatment has not been investigated. There is a 60% lifetime risk of recurrence following the first major depressive episode.^51^ There is also a high chance of relapse among individuals who discontinue antidepressant medication before a 4-month trial period is completed.^52^ Investigating if a similar relapse response can be observed with the KD is important to understand its long-term effects.^53^ For the treatment of epilepsy, the KD (4:1 of fats to carbohydrates and protein) is maintained for a 4-month period. Then, the effectiveness is assessed to decide on the continuation or cessation of the diet.^54^ It is possible that a similar protocol should be implemented for treating MDD. In clinical trials, efficacy is typically assessed as a response to the intervention (i.e. at least a 50% reduction in symptom scales). Effectiveness, however, refers to real-world data, including how much an intervention improves a condition and the long-term adherence rates. A similar framework for assessing the efficacy KD for the treatment of MDD should be employed in future studies, with an additional focus on evaluating effectiveness during follow-up assessments of completed trials. A potential alternative to the fasting or dietary restriction required by the KD that is being explored is the use of exogenous ketone supplements.^55^ Emerging evidence from rodent studies suggests that exogenous ketones supplements can produce rapid and sustainable metabolic changes that mimic those observed during the KD and may produce therapeutic effects for the treatment of various disorders such as anxiety and Alzheimer's disease.^49,56,57^ However, ketone supplements have not yet shown clear efficacy for the treatment of epilepsy, much less for treating MDD, and additional research is necessary to explore this potential alternative to the diet that may provide an option more easily integrated into daily life. Therefore, determining if the KD is feasible for individuals with MDD long-term as well as the effect on symptoms following the discontinuation of the diet is important for clinical application.

### The mechanisms of action remain elusive

Many mechanisms of action of the KD are already known, but those relevant to its antidepressant effects remain unclear. The KD may decrease inflammation by limiting the release of pro-inflammatory cytokines and modulating the expression of pro-inflammatory pathways, an important target for the treatment of MDD.^58,59^ MDD is associated with a constant and low-intensity inflammatory state, which is particularly evident during acute depressive episodes. Inflammatory pathways are a target for many common antidepressants, leading to decreased inflammatory markers upon adherence.^60^

The KD may also affect mitochondrial function, which may be compromised in individuals with MDD.^61^ The mitochondria are organelles responsible for energy generation within cells and regulate brain function through oxidative stress and apoptosis. Increasing the activity of mitochondrial energy synthesis may elicit an antidepressant effect.^61^ KD elicits a metabolic change of the main energy source from glucose to ketones, producing metabolites that impact mitochondrial activity.^62^ This may produce a neuroprotective effect that reduces mitochondrial stress, alleviating symptoms of MDD.^60^

MDD has been associated with insulin resistance (IR), with evidence suggesting a potential link between the conditions. Watson et al. found insulin resistance (IR) to be associated with active major depressive disorder (MDD), but not remitted MDD, suggesting IR is a state-dependent marker.^63^ Moreover, Toba-Oluboka et al.,^64^ reviewed insulin-sensitizing treatments (e.g., pioglitazone, metformin) and observed significant reductions in depressive symptoms, often correlated with improved metabolic measures like glucose tolerance and glycosylated hemoglobin. These associations are relevant because the KD can also improve metabolic conditions and mental health via carbohydrate restriction and glycemic control.^65^ However, Toba-Oluboka emphasized that symptom improvement may be independent of metabolic changes, potentially involving alternative mechanisms like inflammation, neuroinflammation, or cognitive and structural brain changes.^64^ Additionally, the KD is a suggested therapy for IR by improving glycemic control. An open-label, non-randomized, controlled trial observed type-2-diabetes patients (with IR) who began the KD as a possible therapeutic intervention. Participants experienced an improved insulin sensitivity within the first 70 days, with further improvements observed at 1 year, most achieving glycemic control.^66^ Therefore, acknowledging that the relationship between IR and MDD may be a mechanism that the KD acts through to produce antidepressant effects is relevant.

A main target of antidepressant medication is neurotransmitters, such as dopamine, serotonin, glutamate, and Gamma-aminobutyric acid (GABA). SSRIs, for instance, aim to increase serotonin in the brain by decreasing reuptake in the synapse. However, many individuals with MDD do not respond well to antidepressant medications.^67^ Evidence suggests that the KD elicits a decrease in excitatory neurotransmitters (serotonin, dopamine, glutamate) and an increase in inhibitory neurotransmitters (GABA).^68^ Decreasing neuronal hyperactivity in connections associated with depressive symptoms may be one of the mechanisms underlying the antidepressant effect of the KD.^69^ In addition, a suggested mechanism for the therapeutic effects of the KD on epilepsy is the modification of the GABA/Glutamate ratio. A study utilizing an animal model for epilepsy found that the elevated levels of -hydroxybutyric acid, a type of ketone body, induced by the KD, increased the brain GABA/Glutamate ratio. This increase inhibited excitatory neurons, thereby relieving epilepsy symptoms. Due to the similar pathological mechanisms of epilepsy and MDD, a similar mechanism may occur to induce the antidepressant effects of the KD.^70^

Another potential mechanistic link between KD and mood is the bi-directional relationship between gut microbiota and the central nervous system, known as the gut-brain axis.^71^ There is growing evidence for the role of the gut-brain connection in mental disorders. Certain families of bacteria may impact inflammatory pathways and mood.^72,73^ Human and animal studies have found the KD to alter the gut microbiome by decreasing bacterial diversity, abundance, and composition.^74,75^ Opposing evidence suggests increased Bacteroides in the gut microbiome are associated with improved mental health, with the KD eliciting increased levels of Bacteroides. These findings that the KD may improve symptoms of MDD by increasing the levels of positive gut microbiome families.^69^ While there is emerging evidence for a mechanistic role of KD on inflammation, mitochondria, neurotransmitters and the gut-brain axis, future studies are needed to elucidate the mechanistic actions of KD as a psychiatric intervention.

The ketogenic diet (KD) has been shown to be an effective diet for managing obesity,^76^ a condition often linked to depression, although the exact nature of this relationship remains debated.^77^ While weight loss may play a role in the therapeutic effects of KD, a recent review from Dietch and colleagues highlights that significant mood improvements can occur as early as four days into the diet, well before notable weight loss.^44^ This suggests that the benefits of KD are likely driven by ketosis-related mechanisms rather than weight reduction alone.

**Figure 1:**
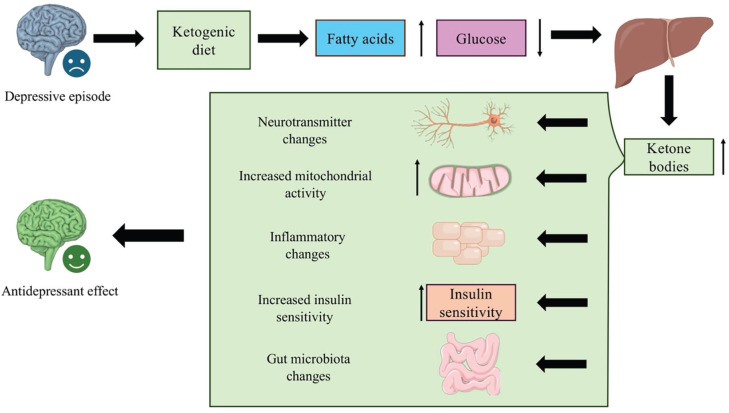
The possible mechanisms of the antidepressant effect of the ketogenic diet. The figure illustrates a brain representative of an individual experiencing a depressive episode. The arrows then depict the individual initiating the ketogenic diet, resulting in an increased presence of fatty acids and a decrease of glucose. This causes the body to enter a state of ketosis, in which the liver produces higher levels of circulating ketone bodies. These ketone bodies may act through five possible mechanisms: modifying neurotransmitter concentration, increasing mitochondria activity, increasing insulin sensitivity, acting on the inflammatory pathways, and modifying the gut microbiome. These possible mechanisms may contribute to the antidepressant effect observed with the ketogenic diet.

### The predictors of response are unknown

Predictors of response to antidepressant interventions have been investigated to understand the complex inter-individual and intra-individual variability in response to treatment.^78,79^ Not all individuals with MDD respond to the same antidepressant treatments. In addition, individuals with depression are often treated with multiple different interventions before improving from their symptoms. The identification of biomarkers of MDD that may be used to determine the most effective treatment of a specific individual is an important research target for the improvement of depression treatment. The CAN-BIND study is a leading effort to identify these biomarkers. Clinical, molecular, and neuroimaging modalities are among the targets investigated to determine predictors.^80^ Functional magnetic resonance imaging (fMRI) studies have identified predictors of response focused on implicit emotional regulation neural circuitry.^79^ For example, an fMRI study revealed an association between greater activity in regions throughout the anterior cingulate/medial prefrontal cortex and a better response to SSRIs.^81^ Another possible approach to identifying predictors is the Personalized Advantage Index (PAI) which combines data obtained on an individual prior to treatment into multivariable prediction models to provide individualized predictions.^78^ We may speculate that if the MDD population is heterogeneous, individuals with metabolic dysfunctions may respond better to the KD treatment. This subpopulation may include individuals with obesity, insulin resistance, and poor diet. In addition, other important demographic predictors could be explored that are likely to impact the sustainability of the diet, such as low-income socioeconomic status or the diagnosis of other psychiatric disorders.

### It is unclear if the KD is feasible for some clinical populations

Feasibility studies of the KD for the treatment of epilepsy have approved the diet for adults. However, studies report a low participant retention rate, often due to side effects, lack of beneficial effects, and the restrictiveness of the diet.^82^ Adherence may be difficult for individuals with MDD due to lack of motivation, changes in appetite, and problematic eating behaviours, which are common within the disorder. However, opposing evidence obtained by a KD trial for bipolar disorder found a relatively high retention rate with successful induction and maintenance of ketosis, suggesting that the KD can be adhered to in a population with serious mental illness if appropriate support is offered.^48^ The exact role of the KD in the treatment of MDD remains unclear. It may be most effective as a combination therapy alongside antidepressants or more beneficial as a monotherapy. Given the preliminary nature of current trials, the KD is typically offered as an adjunct to existing treatment regimens due to ethical reasons. As a result, although the ketogenic diet hold potential to become an alternative to pharmacological treatment or allow a reduction in the dosage or number of medications required, the current data remains insufficient. Therefore, it should be first considered and approved as an adjunctive therapy until further robust evidence supports its standalone efficacy. There is scarce information about follow-up for completed trials and therefore it remains unknown what the termination of the KD does to MDD symptom severity, however none of the previously mentioned studies reported significant complications following the trial. Additionally, feasibility studies are necessary to evaluate the compliance and retention of individuals with MDD following the KD.

### Cost, adherence and social aspects

Another important element to consider for the KD as a treatment is its high cost, primarily due to the increased reliance on protein-rich and high-fat foods. This financial burden can act as a barrier to adopting the diet, particularly for individuals budgeting for family meals.^83^ Additionally, the KD often requires more time for cooking and meal planning, which may not be feasible for some individuals, especially those from lower-income households. This could be partially mitigated by healthcare professionals providing resources such as recipes and cooking techniques to support adherence to the diet. Furthermore, the restrictive nature of the KD may limit socialization during mealtimes, potentially intensifying social isolation, a critical factor contributing to depression.^84^ Negative perceptions of low-carbohydrate diets among the population, may also lead to added stress in social settings or relationships for those on the diet.^85^ Despite these challenges, the KD could offer a viable option for patients who experience inadequate response or poor tolerability to antidepressants, or for those not willing to take pharmacological treatments but are motivated to adopt dietary changes. Preliminary short-term trials indicate a good adherence to the KD, suggesting it may be a feasible option for a subset of patients willing to try nutritional intervention.^9,10,37^

## Conclusion

Research on the efficacy of the KD as a treatment for MDD remains limited. However, emerging studies using animal models and case reports suggest a potential antidepressant effect. The KD's effectiveness for treating childhood epilepsy is well-established, and emerging evidence suggests its feasibility in treating adults as well. Recent studies show beneficial cognitive effects of the KD, revealing improvements in working memory and cognitive performance.^29,30,32,33^ Should the KD be an effective treatment for MDD, it would provide an attractive alternative or adjunct treatment for patients who do not respond adequately to antidepressant treatments. Establishing the clinical effectiveness of the KD will expand the scope of recognized clinical interventions for psychiatric disorders.

Given that MDD is suggested to have a metabolic component,^86^ and the established capacity of the KD to modulate metabolism,^62^ it is possible that subpopulations exhibiting metabolic dysfunction may have better responses to this form of intervention.^87^ An essential consideration in the treatment of MDD is the feasibility of the approach. Should the KD be found to be effective for MDD, the same clinical support that individuals with epilepsy receive have the potential to be adopted by insurance carriers and can facilitate effective treatment despite the challenges seen in this population. It is important to note that most studies in this area are preclinical or case studies, limiting the ability to draw conclusions on the diet's safety and efficacy. Larger, controlled clinical studies are imperative to fill these gaps of knowledge and to further progress the establishment of the KD as a viable treatment for MDD. This article has summarized the current literature and gaps in knowledge with the goal to promote attention and further investigation into this potential new avenue of antidepressant treatment (Box 1).

**Box 1:** The KD as a treatment for MDD

What we know…	What we want to know…
The KD is overall safe for most individuals. Common short-term symptoms (i.e. headaches, nausea, constipation) present during the initiation of the diet and tend to resolve themselves. The KD is a well-established treatment of refractory epilepsy. It has been proven to produce an anti-convulsant effect in children and adults with epilepsy. The KD has demonstrated its ability to produce favourable cognitive effects. Preclinical studies suggest a long-term improvement of cognitive function. Studies have also observed improvements in cognitive performance among individuals with neuropsychiatric disorders following the implementation of the diet. Emerging research suggest that the KD elicits antidepressant and mood stabilizing effects in individuals with MDD. In animal studies, reductions in depressive behaviours have been observed following the administration of the KD. In addition, case studies have documented reductions in depression symptom severity reduction in individuals with MDD on the KD.	The therapeutic window of the prospective treatment (i.e. the level of ketones in the body necessary to induce an antidepressant effect). Epilepsy research suggests that less restrictive alternatives to the classic KD may be equally as effective in producing an anticonvulsant effect. The long-term effectiveness of the KD as a treatment for MDD has yet to be determined. Relapse following the cessation of antidepressant medication is very common among individuals with MDD. Perhaps a similar protocol to the treatment of epilepsy could be implemented for MDD, which involves a gradual modification of the KD to include more protein and/or carbohydrates and/or less fat, making the diet more enjoyable and sustainable. The mechanism(s) of action of the antidepressant effects of the KD remains unknown. Various physiological pathways have been proposed to be involved in MDD: inflammatory pathways, insulin sensitivity, mitochondrial energy generation, neurotransmitter availability and changes in the gut microbiome. The predictors of response of any antidepressant intervention are being investigated. Further investigation of these predictors may identify subpopulations that are highly responsive to the KD as an antidepressant. Populations with metabolic dysfunction (e.g. obesity, insulin resistance, poor diet) may be more susceptible to the treatment intervention. It remains unclear whether the KD is a feasible intervention for individuals with MDD. It may be challenging for individuals experiencing depressive episodes to abide to a strict dietary regime such as the KD. Additional feasibility testing should be done prior to clinical application of the treatment.

## Data Availability

The data that support this study are available in the body of the paper.
